# Hippo cooperates with p53 to regulate lung airway mucous cell metaplasia

**DOI:** 10.1242/dmm.052074

**Published:** 2024-11-18

**Authors:** Jiangying Liu, Dan Luo, Haidi Huang, Rongzi Mu, Jianghong Yuan, Ming Jiang, Chuwen Lin, Honggang Xiang, Xinhua Lin, Haihan Song, Yongchun Zhang

**Affiliations:** ^1^ State Key Laboratory of Microbial Metabolism, Joint International Research Laboratory of Metabolic and Developmental Sciences, Inner Mongolia Research Institute, Shenzhen Research Institute, Sheng Yushou Center of Cell Biology and Immunology, School of Life Sciences and Biotechnology, Shanghai Jiao Tong University, Shanghai 200240, China; ^2^ Center for Genetic Medicine, The Fourth Affiliated Hospital, Zhejiang University School of Medicine, Hangzhou 310030, Zhejiang, China; ^3^ Department of Histology and Embryology, School of Medicine, Shenzhen Campus of Sun Yat-Sen University, Shenzhen 518107, Guangdong, China; ^4^ Department of General Surgery, Pudong New Area People's Hospital, Shanghai 201299, China; ^5^ State Key Laboratory of Genetic Engineering, School of Life Sciences, Greater Bay Area Institute of Precision Medicine (Guangzhou), Zhongshan Hospital, Fudan University Shanghai, Shanghai 200438, China; ^6^ Central Lab, Shanghai Key Laboratory of Pathogenic Fungi Medical Testing, Shanghai Pudong New Area People's Hospital, Shanghai 201299, China; ^7^ Department of Immunology, DICAT National Biomedical Computation Centre, Vancouver, BC V6B 5A6, Canada

**Keywords:** Club cell, Goblet cell, Mucous cell metaplasia, Hippo signaling, p53

## Abstract

Airway mucous cell metaplasia is a significant feature of many chronic airway diseases, such as chronic obstructive pulmonary disease, cystic fibrosis and asthma. However, the mechanisms underlying this process remain poorly understood. Here, we employed *in vivo* mouse genetic models to demonstrate that Hippo and p53 (encoded by *Trp53*) cooperate to modulate the differentiation of club cells into goblet cells. We revealed that ablation of *Mst1* (*Stk4*) and *Mst2* (*Stk3*), encoding the core components of Hippo signaling, significantly reduces mucous metaplasia in the lung airways in a lipopolysaccharide (LPS)-induced lung inflammation murine model while promoting club cell proliferation in a Yap (Yap1)-dependent manner. Additionally, we showed that deleting Mst1/2 is sufficient to suppress p53 deficiency-mediated goblet cell metaplasia. Finally, single-cell RNA-sequencing analysis revealed downregulation of YAP and p53 signaling in goblet cells in human airways. These findings underscore the important role of Hippo and p53 signaling in regulating airway mucous metaplasia.

## Teaser

**Summary:** This work employs mouse genetic models to demonstrate that Hippo signaling deficiency suppresses lung airway mucous cell metaplasia induced by lipopolysaccharide or p53 deletion.

## INTRODUCTION

In the pulmonary conducting airways, goblet cells secrete mucins to protect the lung by assisting in the clearance of toxic substances and cellular debris (Curran and Cohn, 2010; Whitsett, 2018). However, excessive mucous cell metaplasia, which occurs in many chronic airway diseases, such as chronic obstructive pulmonary disease (COPD), cystic fibrosis and asthma, can lead to airway obstruction and even mortality (Curran and Cohn, 2010). The airway epithelium is primarily composed of club cells, ciliated cells, basal cells, goblet cells and a rare population of neuroendocrine cells (Hogan et al., 2014; Morrisey and Hogan, 2010; Rawlins et al., 2009). Under normal conditions, most club cells are non-proliferative, and goblet cells represent only a minor population of the epithelial cells. However, following injury induced by various insults such as chemicals, infection or allergens, club cells can proliferate and differentiate into ciliated cells and goblet cells to repair the airway epithelium (Chen et al., 2009; Hicks-Berthet et al., 2021; Rawlins et al., 2009). Studies have demonstrated that inflammation during chronic injury can result in goblet cell hyperplasia and mucous hypersecretion, mediated by cytokines including IL13 and IL1 (Fujisawa et al., 2011; Kuperman et al., 2002). The delivery of bacterial lipopolysaccharide (LPS), which is present in air pollution and dust, into the mouse airways to elicit inflammation and injury has been widely used as an *in vivo* model to study goblet cell metaplasia (Glasser et al., 2013; Yanagihara et al., 2001). Moreover, recent studies have revealed that intrinsic signaling regulation within club cells also plays a critical role in goblet cell differentiation. For example, Spdef is a transcription factor that has been shown to be both required and sufficient for driving pulmonary goblet cell differentiation (Chen et al., 2009; Park et al., 2007). Additionally, signaling pathways such as Notch and EGFR signaling promote, whereas KDR signaling represses, the differentiation process (Danahay et al., 2015; Guha et al., 2014; Guseh et al., 2009; Jiang et al., 2021; Tyner et al., 2006). However, the molecular mechanisms underlying goblet cell metaplasia remain far from being fully understood.

Hippo signaling is an important signaling pathway that regulates multiple biological processes, including cell proliferation and differentiation, and is involved in the development of multiple diseases (Yu et al., 2015). The kinases Mst1 (Stk4) and Mst2 (Stk3) are the core components of Hippo signaling (Yu et al., 2015). When Hippo signaling is activated, Mst1/2 phosphorylate the kinases Lats1/2, which in turn phosphorylate the downstream transcriptional co-factors Yap (Yap1)/Taz. The phosphorylation leads to the retention of Yap/Taz in the cytoplasm or their degradation (Yu et al., 2015; Zheng and Pan, 2019). In contrast, the inactivation of Hippo signaling results in the dephosphorylation and stabilization of Yap/Taz, allowing their translocation into the nucleus to transactivate downstream target genes by cooperating with transcription factors Tead1-4 (Yu et al., 2015; Zheng and Pan, 2019). Given the complexity of Hippo signaling transduction, elucidating the function of each signaling component in regulating different biological processes, such as cell fate determination, is of significance.

Prior studies have demonstrated that Hippo signaling regulates the function of epithelial cells in various tissues. For example, knockout of Mst1/2 promotes epithelial cell proliferation in the liver and intestine, leading to epithelial hyperplasia (Zhou et al., 2009, 2011). Our recent research also showed that Hippo signaling restrains basal cell proliferation in the esophageal epithelium and cooperates with p53 (encoded by *Trp53*) to repress squamous cell carcinoma initiation (Jiang et al., 2024). Similarly, in the lung conducting airways, loss of Mst1/2 or overexpression of Yap in the airway epithelium results in basal cell hyperplasia (Lange et al., 2015; Volckaert et al., 2017; Zhao et al., 2014), while loss of Yap in club cells leads to goblet cell hyperplasia (Hicks-Berthet et al., 2021). These studies highlight multiple roles of Hippo signaling in the regulation of epithelial cell function in the airways. However, the role of Hippo signaling in goblet cell differentiation has not been determined *in vivo*. Moreover, p53 has been shown to repress goblet cell metaplasia in the airways (Chand et al., 2014). Whether Hippo cooperates with p53 to regulate goblet cell differentiation remains undetermined.

In this study, we first utilized an LPS-induced lung inflammation mouse model to reveal that deletion of *Mst1/2* largely reduces goblet cell metaplasia in the airway epithelium. Furthermore, through immunostaining analyses, we showed that *Mst1/2* deficiency represses the differentiation of club cells into goblet cells while promoting club cell proliferation. Additional genetic studies in mice indicated that *Mst1/2* deficiency regulates goblet cell differentiation in a Yap-dependent manner. Moreover, *Mst1/2* ablation was sufficient to repress goblet cell metaplasia induced by *p53* deficiency. Finally, single-cell RNA-sequencing analysis of human airway epithelial cells revealed downregulation of Yap and p53 signaling in goblet cells compared to club cells. Collectively, our findings underscore the crucial role of Hippo signaling in controlling club cell fate and airway mucous metaplasia.

## RESULTS

### Deletion of Mst1/2 inhibits lung airway LPS-induced mucous cell hyperplasia

**Fig. 1.**
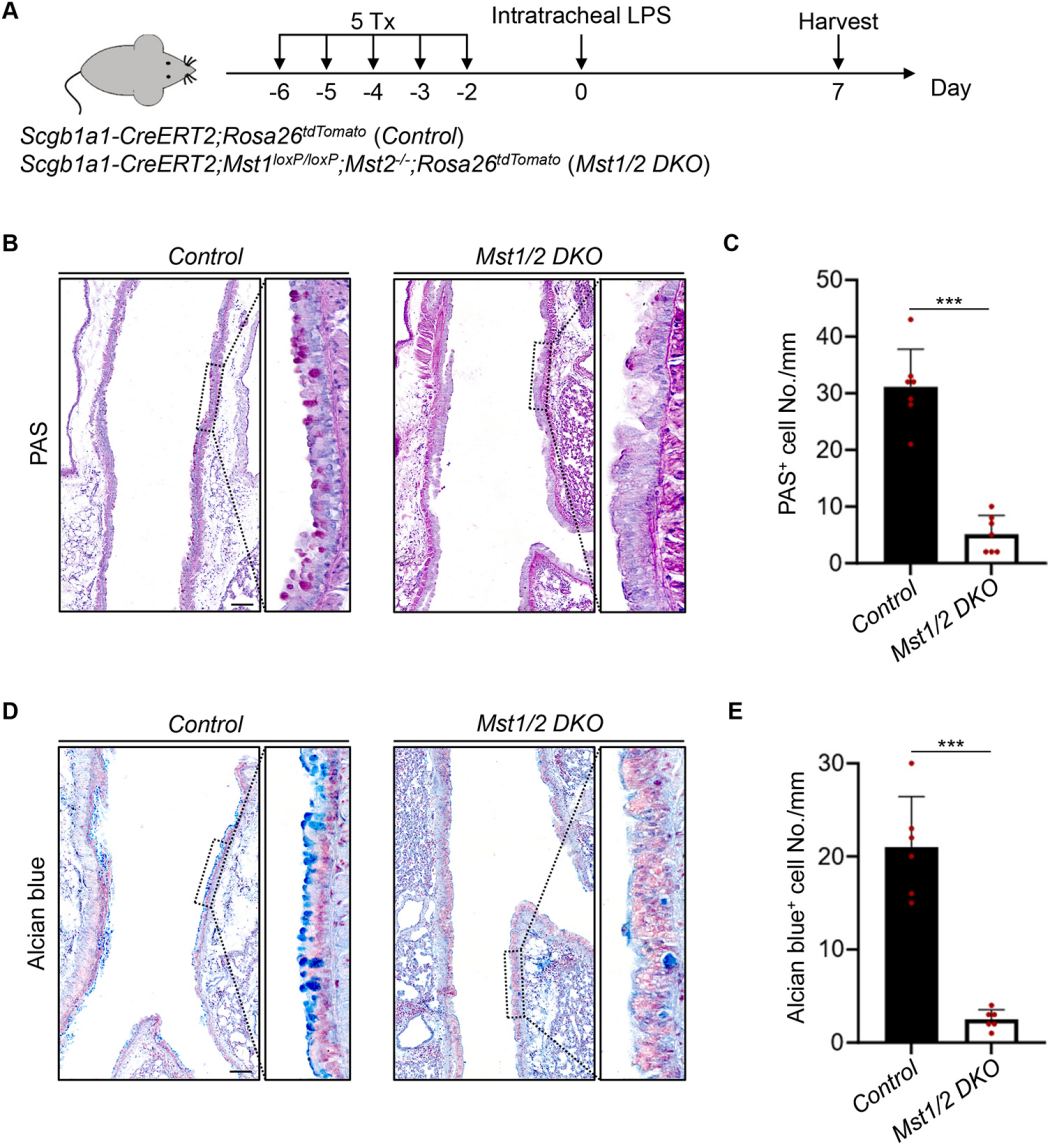
**Deletion of Mst1/2 inhibits lung airway lipopolysaccharide (LPS)-induced mucous cell hyperplasia.** (A) Schematic diagram of the LPS treatment procedure. Tamoxifen (Tx) administration to *Scgb1a1-CreERT2; Mst1^loxP/loxP^; Mst2^−/−^;Rosa26^tdTomato^* [*Mst1/2* double knockout (DKO)] mice leads to the deletion of *Mst1/2* in club cells of the airway epithelium. *Scgb1a1-CreERT2;Rosa26^tdTomato^* (control) mice were used as control. (B,C) Periodic acid–Schiff (PAS) staining (B) and quantification of PAS^+^ cell number (C) in the airway epithelium. Note the decrease in PAS^+^ cells in the *Mst1/2* DKO mutants. Data represent mean±s.d. (*n*=7 per genotype). ***P<0.001, by unpaired, two-tailed Student's *t*-test. Scale bar: 100 μm. (D,E) Alcian Blue staining (D) and quantification of Alcian Blue^+^ cell number (E) in the airway epithelium of control and *Mst1/2* DKO mice. Note the decrease in Alcian Blue^+^ cells in the *Mst1/2* DKO mice. Data represent mean±s.d. (*n*=6 per genotype). ***P<0.001, by unpaired, two-tailed Student's *t*-test. Scale bar: 100 μm.

To determine the role of Mst1/2 in the cell fate determination of club cells, we first generated *Scgb1a1-CreERT2;Mst1^loxP/loxP^;Mst2^−/−^* mice and administered them with five consecutive doses of tamoxifen to induce *Mst1/2* double knockout (DKO) in the airway club cells, using *Scgb1a1-CreERT2* mice as control (Fig. 1A). Next, bacterial LPS was delivered via intratracheal instillation to induce airway inflammation (Fig. 1A). Seven days after LPS treatment, periodic acid–Schiff (PAS) staining, which detects neutral mucus, was performed on the tissues. The staining showed induction of PAS^+^ mucous cell hyperplasia in the airway epithelium in the LPS-treated group, but few PAS^+^ cells in the vehicle-treated group (Fig. 1B; Fig. S1A,B**)**. Notably. *Mst1/2* deletion led to a large reduction in PAS^+^ mucous cells compared to the control (Fig. 1B,C). We also performed Alcian Blue staining, which detects acidic mucins in tissues. Consistently, Alcian Blue^+^ cells were significantly reduced in the airway epithelium upon *Mst1/2* deletion (Fig. 1D,E). Taken together, these results demonstrate that the loss of Hippo signaling represses mucous metaplasia in the airway epithelium.

### Mst1/2 deficiency represses goblet cell differentiation but promotes the proliferation of club cells in LPS-induced inflammation

**Fig. 2.**
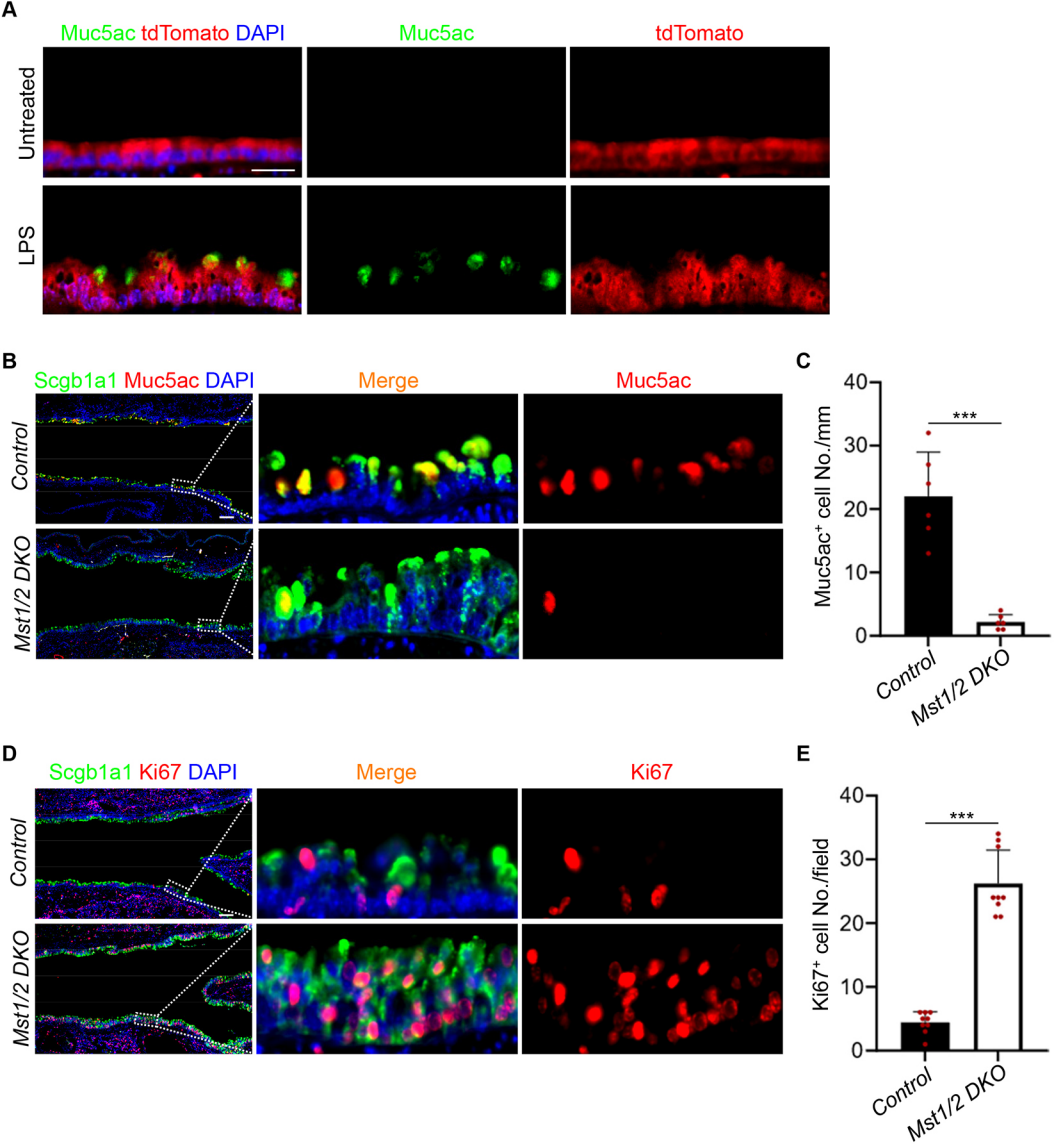
**Mst1/2 deficiency represses goblet cell differentiation but promotes the proliferation of club cells in LPS-induced inflammation.** (A) Immunofluorescence (IF) staining of goblet cell marker Muc5ac and lineage tracing marker tdTomato in the airway epithelium of untreated and LPS-treated *Scgb1a1-CreERT2;Rosa26^tdTomato^* mice. The staining indicates that goblet cells are derived from club cells. Scale bar: 25 μm. (B) IF staining of club cell marker Scgb1a1 and goblet cell marker Muc5ac in the airway epithelium of control and *Mst1/2* DKO mice. Scale bar: 100 μm. (C) Quantification of Muc5ac^+^ cells in the airway epithelium. Note the decrease in the Muc5ac^+^ cells in the *Mst1/2* DKO mutants. Data represent mean±s.d. (*n*=6 per genotype). ***P<0.001, by unpaired, two-tailed Student's *t*-test. (D) IF staining of club cell marker Scgb1a1 and proliferation marker Ki67 (Mki67) in the airway epithelium. Scale bar: 100 μm. (E) Quantification of Ki67^+^ proliferating cells in the airway epithelium. Note the increase in proliferating cells in *Mst1/2* DKO mutants. Data represent mean±s.d. (*n*=9 per genotype). ***P<0.001, by unpaired, two-tailed Student's *t*-test. Control, *Scgb1a1-CreERT2;Rosa26^tdTomato^*; *Mst1/2* DKO, *Scgb1a1-CreERT2;Mst1^loxP/loxP^;Mst2^−/−^;Rosa26^tdTomato^*. DAPI, 4′,6-diamidino-2-phenylindole.

Prior studies have shown that club cells serve as progenitor cells of goblet cells in the intrapulmonary airway epithelium (Chen et al., 2009). We conducted lineage tracing using *Scgb1a1-CreERT2;Rosa26^tdTomato^*, in which club cells and their progenies express tdTomato upon tamoxifen treatment. Our results showed that all Muc5ac^+^ goblet cells were derived from club cells following LPS treatment (Fig. 2A). Further immunostaining revealed that the differentiation of club cells into Muc5ac^+^ goblet cells was significantly reduced by *Mst1/2* deletion (Fig. 2B,C). However, *Mst1/2* DKO highly increased the proliferation of club cells after LPS treatment (Fig. 2D,E), which is consistent with a role for Hippo signaling in repressing airway epithelial proliferation during homeostasis (Lange et al., 2015; Volckaert et al., 2017). Similarly, we also observed an increase in club cell proliferation in our *Mst1/2* DKO mutants under homeostatic conditions (Fig. S2A-C). Overall, these findings support that the loss of Hippo signaling suppresses the differentiation of club cells into goblet cells while promoting their proliferation during LPS-induced inflammation.

### Mst1/2 deficiency suppresses mucous metaplasia but promotes club cell proliferation through Yap during LPS-induced inflammation

**Fig. 3.**
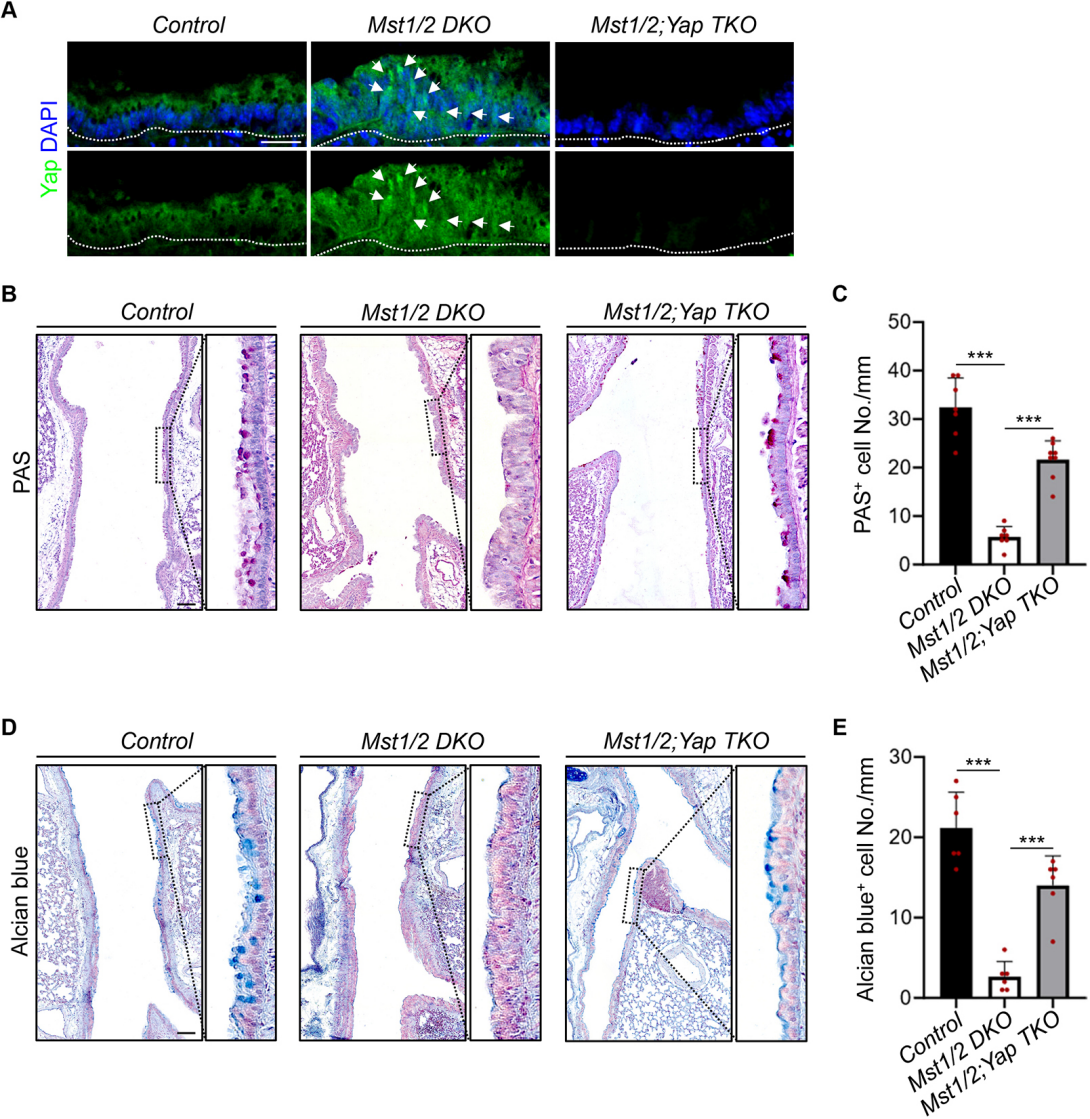
**Mst1/2 deficiency suppresses mucous metaplasia through Yap during LPS-induced inflammation.** (A) IF staining of Yap in the airway epithelium of control, *Mst1/2* DKO and *Mst1/2;Yap* TKO mice. Note the increased nuclear localization of Yap (white arrows) in *Mst1/2* DKO mutants compared to the control and absence of expression of Yap in *Mst1/2;Yap* TKO mutants. The white dotted lines denote the airway epithelium at the top and the mesenchyme at the bottom. Scale bar: 25 µm. (B) PAS staining of the airway epithelium 7 days post-LPS treatment. Scale bar: 100 μm. (C) Quantification of PAS^+^ cell number in the airway epithelium. Note the decrease in PAS^+^ cells but a significant increase upon *Yap* deletion. Data represent mean±s.d. (*n*=7 for control and *Mst1/2* DKO, *n*=8 for *Mst1/2;Yap* TKO). ***P<0.001, by unpaired, two-tailed Student's *t*-test. (D) Alcian Blue staining in the airway epithelium 7 days post-LPS treatment. Scale bar: 100 μm. (E) Quantification of Alcian Blue^+^ cell number in the airway epithelium. Data represent mean±s.d. (*n*=6 per genotype). ***P<0.001, by unpaired, two-tailed Student's *t*-test. Control, *Scgb1a1-CreERT2*; *Mst1/2* DKO, *Scgb1a1-CreERT2;Mst1^loxP/loxP^;Mst2^−/−^*; *Mst1/2;Yap* TKO, *Scgb1a1-CreERT2;Mst1^loxP/loxP^;Mst2^−/−^;Yap^loxP/loxP^*.

**Fig. 4.**
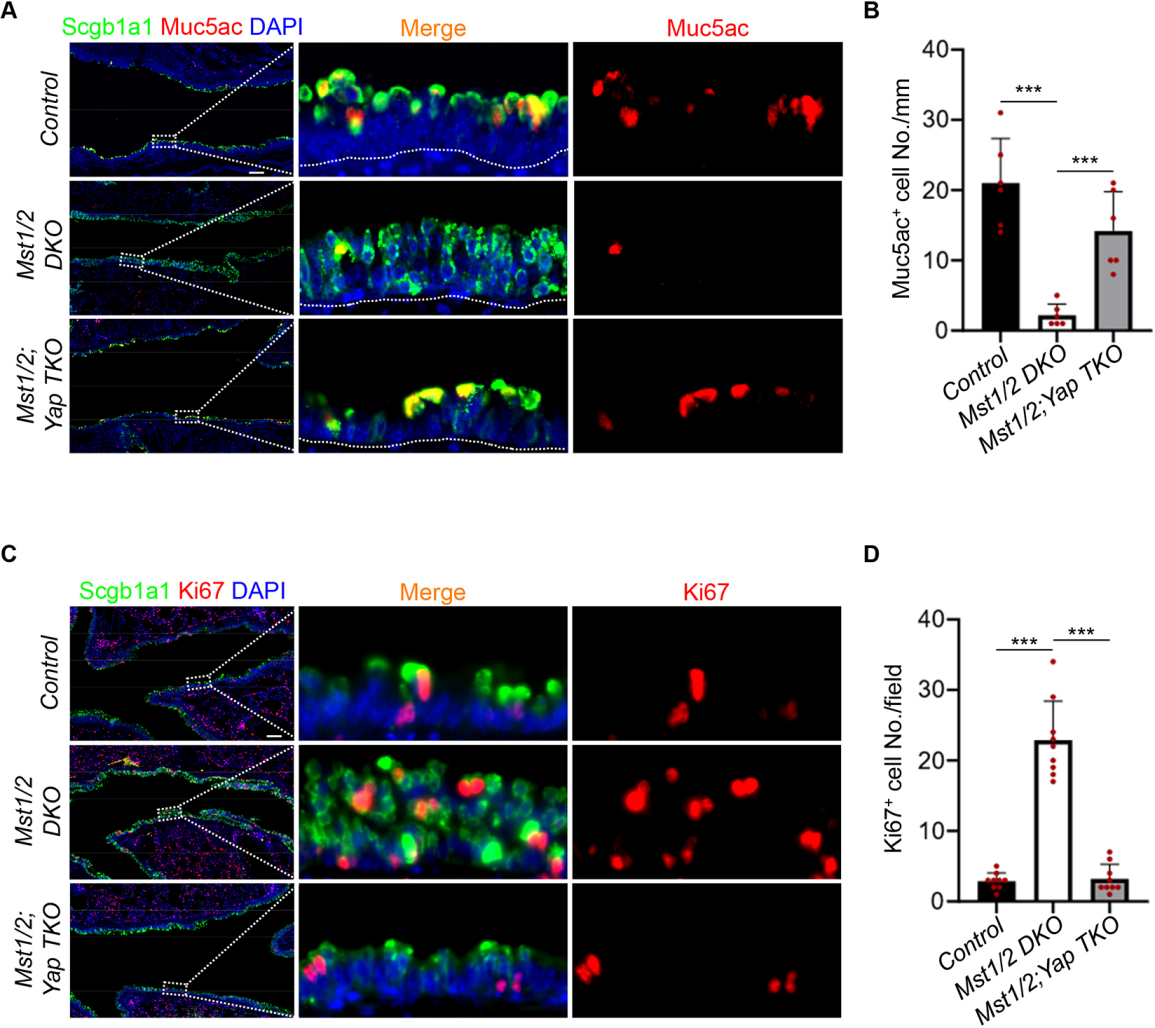
**Mst1/2 deficiency suppresses mucous metaplasia but promotes club cell proliferation through Yap during LPS-induced inflammation.** (A) IF staining of club cell marker Scgb1a1 and goblet cell marker Muc5ac in the airway epithelium 7 days post-LPS treatment. The white dotted lines denote the airway epithelium at the top and the mesenchyme at the bottom. Scale bar: 100 μm. (B) Note the decreased Muc5ac^+^ goblet cells in *Mst1/2* DKO mice but increased Muc5ac^+^ goblet cells with *Yap* deletion. Data represent mean±s.d. (*n*=6 per genotype). ***P<0.001, by unpaired, two-tailed Student's *t*-test. (C) IF staining of club cell marker Scgb1a1 and cell proliferation marker Ki67 in the airway epithelium of control, *Mst1/2* DKO and *Mst1/2;Yap* TKO mice after LPS treatment. Scale bar: 100 μm. (D) Note the increased Ki67^+^ cells in *Mst1/2* DKO mice but reduced proliferating (Ki67^+^) cells with *Yap* deletion. Data represent mean±s.d. (*n*=9 per genotype). ***P<0.001, by unpaired, two-tailed Student's *t*-test. Control, *Scgb1a1-CreERT2*; *Mst1/2* DKO, *Scgb1a1-CreERT2;Mst1^loxP/loxP^;Mst2^−/−^*; *Mst1/2;Yap* TKO, *Scgb1a1-CreERT2;Mst1^loxP/loxP^;Mst2^−/−^;Yap^loxP/loxP^*.

Mst1/2 are well-known upstream negative regulators of Yap signaling by preventing its nuclear entry (Yu et al., 2015). Immunostaining revealed that the nuclear localization of Yap is elevated upon *Mst1/2* deletion, while Yap is absent upon *Yap* deletion (Fig. 3A). To determine whether Mst1/2 regulate the function of club cells in a Yap-dependent manner, we ablated *Yap* in *Mst1/2*-deficient mice. This significantly upregulated the PAS^+^ and Alcian Blue^+^ mucous epithelial cells within the airway epithelium (Fig. 3B-E). Further immunostaining analysis revealed a consistent increase in Muc5ac^+^ goblet cells with *Yap* deletion (Fig. 4A,B). Additionally, *Yap* ablation significantly reduced the increase in proliferating club cells observed with *Mst1/2* deletion (Fig. 4C,D). Taken together, these results demonstrate that *Mst1/2* deficiency represses mucous metaplasia and enhances club cell proliferation in a Yap-dependent manner.

### Mst1/2 deletion represses mucous metaplasia induced by p53 deficiency

**Fig. 5.**
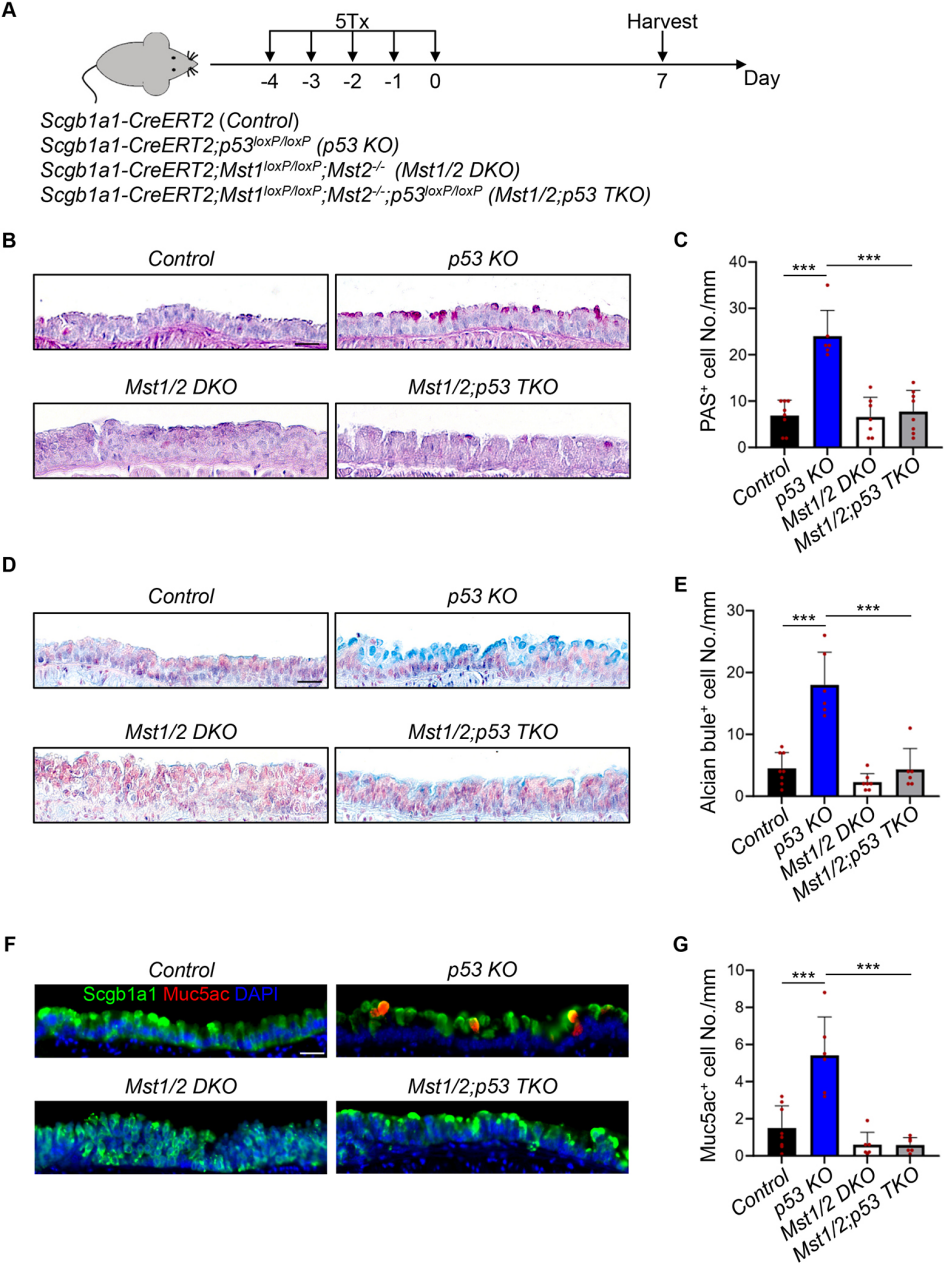
**Mst1/2 deletion represses mucous metaplasia induced by p53 deficiency.** (A) Schematic diagram of the experimental strategies. Control, *Scgb1a1-CreERT2*; *p53* KO, *Scgb1a1-CreERT2;p53^loxP/loxP^*; *Mst1/2* DKO, *Scgb1a1-CreERT2;Mst1^loxP/loxP^;Mst2^−/−^*; *Mst1/2;p53* TKO, *Scgb1a1-CreERT2;Mst1^loxP/loxP^;Mst2^−/−^;p53^loxP/loxP^.* (B) PAS staining in the airway epithelium. Scale bar: 25 μm. (C) Quantification of PAS^+^ cells in the airway epithelium. Note that the increase in PAS^+^ cells upon *p53* deletion was repressed by *Mst1/2* deficiency. Data represent mean±s.d. (*n*=8 for control, *n*=6 for *p53* KO, *n*=7 for *Mst1/2* DKO and *n*=8 for *Mst1/2;p53 TKO*). ***P<0.001, by unpaired, two-tailed Student's *t*-test. (D) Alcian Blue staining in the airway epithelium. Scale bar: 25 μm. (E) Quantification of Alcian Blue^+^ cells in the airway epithelium. Note that the increase in Alcian Blue^+^ cells upon *p53* deletion was repressed by *Mst1/2* deficiency. Data represent mean±s.d. (*n*=8 for control, *n*=6 for *p53* KO, *n*=7 for *Mst1/2* DKO and *n*=6 for *Mst1/2;p53* TKO). ***P<0.001, by unpaired, two-tailed Student's *t*-test. (F) IF staining of club cell marker Scgb1a1 and goblet cell marker Muc5ac in the airway epithelium. Scale bar: 25 μm. (G) Quantification of Muc5ac^+^ cells in the airway epithelium. Note that the increase in Muc5ac^+^ goblet cells upon *p53* deletion was repressed by *Mst1/2* deficiency. Data represent mean±s.d. (*n*=8 for control, *n*=6 for *p53* KO, *n*=6 for *Mst1/2* DKO and *n*=6 for *Mst1/2;p53* TKO). ***P<0.001, by unpaired, two-tailed Student's *t*-test.

Previous studies have reported that p53 loss in club cells leads to mucous hyperplasia in the airways (Chand et al., 2014). We further investigated whether Hippo signaling plays a role in *p53* deletion-mediated mucous metaplasia (Fig. 5A). Consistent with prior findings (Chand et al., 2014), *p53* loss resulted in an increase in PAS^+^ and Alcian Blue^+^ mucous cells in the homeostatic condition (Fig. 5B-E). Immunostaining of the tissues further revealed an increase in Muc5ac^+^ goblet cells upon *p53* deletion, whereas *Mst1/2* deletion significantly reduced the goblet cell numbers (Fig. 5F,G). Therefore, these findings indicate that Hippo signaling is required for *p53* deficiency-mediated mucous hyperplasia.

### Single-cell RNA-sequencing analysis of human airway epithelial cells reveals downregulation of Yap and p53 signaling during goblet cell differentiation

**Fig. 6.**
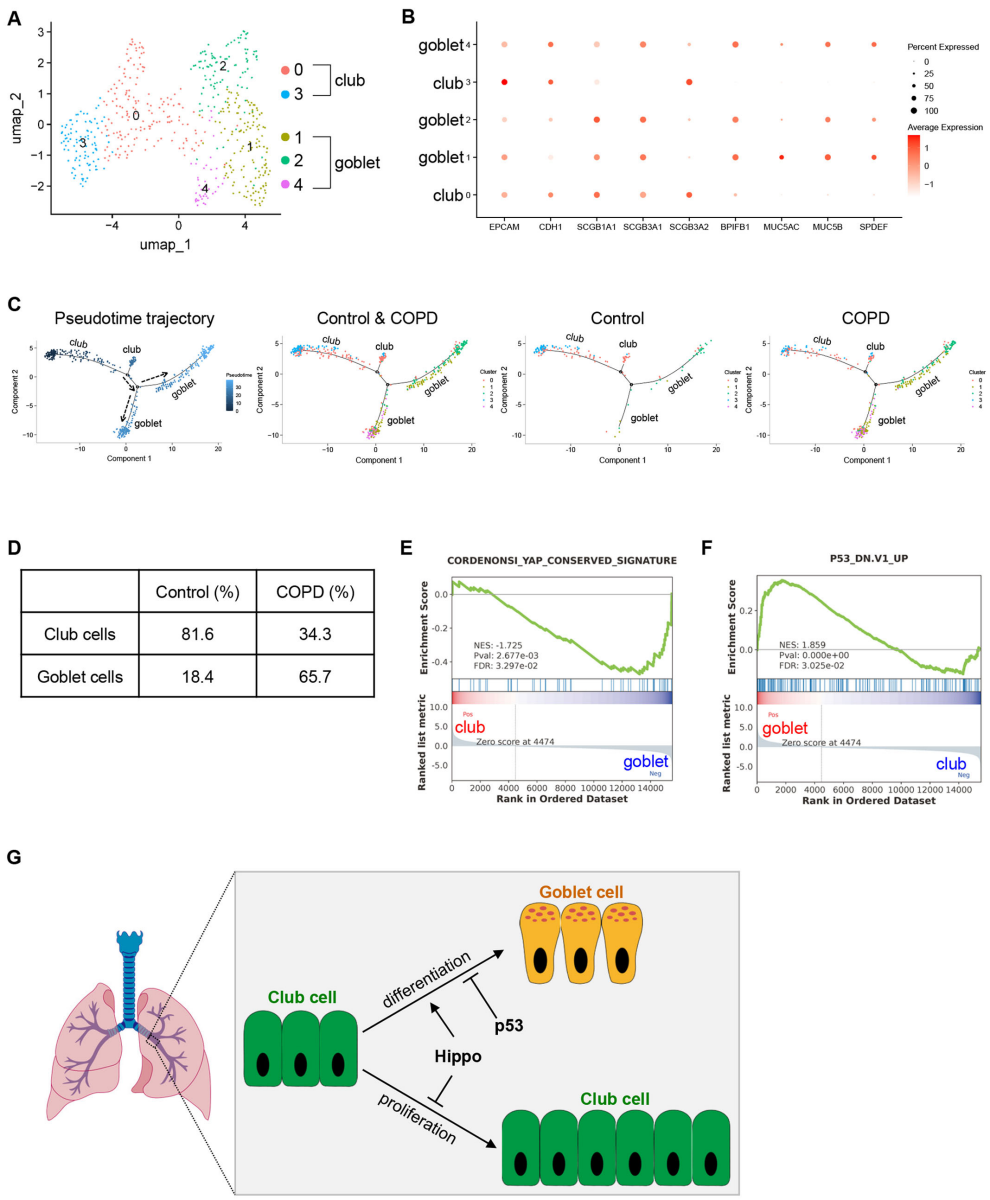
**Single-cell RNA-sequencing analysis of human airway epithelial cells reveals downregulation of Yap and p53 signaling during goblet cell differentiation.** (A) Uniform manifold approximation and projection (UMAP) plot of single-cell RNA-sequencing data on club and goblet cells. (B) Dot plot depicting expression of epithelial cell markers *EPCAM* and *CDH1* (E-cadherin); club cell markers *SCGB1A1*, *SCGB3A1* and *SCGB3A2*; and goblet cell markers *BPIFB1*, *MUC5AC*, *MUC5B* and *SPDEF*. (C) Pseudotime trajectory analysis of different cell types. (D) Percentage of each type of cell in the non-COPD (control) and chronic obstructive pulmonary disease (COPD) patient samples. Note that the percentage of goblet cells is higher in COPD patient samples than in control samples. (E) Gene set enrichment analysis (GSEA) indicates the downregulation of YAP signatures in goblet cells. (F) GSEA indicates the upregulation of the p53-negatively regulated signature in goblet cells. ‘P53_DN.V1_UP’ annotates a gene signature that is upregulated with p53 mutations. FDR, false discovery rate; NES, normalized enrichment score; Pval, *P*-value. (G) The model shows that Hippo signaling represses the proliferation of club cells and cooperates with p53 to modulate goblet cell differentiation.

To investigate the clinical implications of our findings, we analyzed single-cell RNA-sequencing data from a previous study, including nine COPD and seven non-COPD (control) samples (Watanabe et al., 2022). We focused on the club cells and goblet cells. Uniform manifold approximation and projection (UMAP) analysis revealed two clusters of club cells with high expression of SCGB1A1, SCGB3A1 and SCGB3A2 (Guha et al., 2012; Reynolds et al., 2002), and three clusters of goblet cells with high expression of BPIFB1, MUC5AC, MUC5B and SPDEF (Bingle and Craven, 2002; Chen et al., 2009; Okuda et al., 2019; Park et al., 2007) (Fig. 6A,B). Pseudotime trajectory analysis showed that club cells serve as progenitors of goblet cells (Fig. 6C), which is consistent with the mouse lineage tracing study and prior studies (Chen et al., 2009) (Fig. 2A). Moreover, COPD samples had a higher percentage of goblet cells than did control samples (control, 18.4%; COPD, 65.7%) (Fig. 6D). Significantly, gene set enrichment analysis (GSEA) indicated downregulation of YAP and p53 signatures in the goblet cells compared to club cells (Fig. 6E,F). These results highlight the significant clinical implications of Hippo and p53 signaling in airway goblet cell differentiation.

## DISCUSSION

In this study, we revealed that Hippo cooperates with p53 to regulate the function of club cells within the mouse airway epithelium (Fig. 6G). We found that depletion of *Mst1/2* in club cells significantly reduces goblet cell metaplasia while promoting club cell proliferation in a mouse model of LPS-induced lung injury. Further genetic studies in mice demonstrated that *Mst1/2* deficiency represses goblet cell differentiation and promotes the proliferation of club cells in a Yap-dependent manner. Additionally, we showed that Mst1/2 are required for *p53* loss-mediated goblet cell hyperplasia. Single-cell RNA-sequencing data indicated downregulation of YAP and p53 signaling during goblet cell differentiation in the human airways.

Despite prior studies showing that *Yap/Taz* deletion promotes goblet cell differentiation (Hicks-Berthet et al., 2021), the *in vivo* function of the essential Hippo signaling kinase components, Mst1/2, has not been determined. Here, we employed an LPS-induced pulmonary inflammation mouse model to reveal that deleting *Mst1/2* results in significant reduction in the mucous cells derived from club cells in the conducting airways. Immunostaining studies showed that the loss of Mst1/2 enhances the nuclear localization of Yap, consistent with the negative regulation of Yap signaling by Mst1/2 (Yu et al., 2015; Zheng and Pan, 2019). Subsequently, we generated *Mst1/2;Yap* triple knockout (TKO) mice to demonstrate that *Mst1/2* deficiency-mediated reduction in goblet cell metaplasia relies on Yap. Yap and Tead proteins have been shown to bind to the promoter of Spedf, an essential transcription factor for goblet cell differentiation, and repress its expression (Chen et al., 2009; Hicks-Berthet et al., 2021; Park et al., 2007). Therefore, it is possible that the increased nuclear levels of Yap upon *Mst1/2* deletion led to a decrease in Spdef-mediated goblet cell differentiation. Taken together, these findings support the role of Hippo signaling in promoting mucous cell metaplasia in an *in vivo* setting.

Additionally, studies have shown that proliferation of airway epithelial cells is increased with *Mst1/2* deletion (Volckaert et al., 2017). Consistently, we observed that *Mst1/2* ablation also leads to an increase in the proliferation of club cells, which has been shown to serve as important epithelial stem cells for airway epithelial repair during LPS-induced injury (Rawlins et al., 2009). This proliferative increase was reserved by *Yap* ablation. Our findings underscore the significant role of Hippo signaling in restraining the proliferation of these stem cells during injury.

p53 is a tumor suppressor involved in repressing the initiation and progression of cancers such as lung and gastric cancer (Chand et al., 2014; Sethi et al., 2020; Stiewe, 2007). Our recent studies revealed that p53 cooperates with Hippo signaling to inhibit foregut squamous cell carcinoma (Jiang et al., 2024). Similarly, other studies have demonstrated that deletion of *p53* promotes liver carcinogens induced by the loss of *Mst1/2* (Zhang et al., 2017). It is of significance to investigate whether tumors will also initiate in the airways of *Mst1/2;p53* TKO (*Scgb1a1-CreERT2;Mst1^loxP/loxP^;Mst2^−/−^;p53^loxP/loxP^*) mice in future studies. In addition to its critical role in tumorigenesis, p53 regulates the differentiation of multiple tissues, such as by promoting the terminal differentiation of mouse embryonic stem cells and skin keratinocytes (Almog and Rotter, 1997; Lin et al., 2005; Woodworth et al., 1993). In the lung airways, *p53* deficiency promotes goblet cell hyperplasia. To determine whether Hippo interacts with p53 to regulate goblet cell differentiation in the airways, we generated club cell-specific *p53* deletion mice. Our results showed that loss of *p53* leads to goblet cell metaplasia, while ablating *Mst1/2* is sufficient to reverse such a process. Therefore, our studies demonstrate that Hippo is required for *p53* loss-mediated mucous cell metaplasia. These findings are significant as they reveal the essential role of Hippo signaling in goblet cell metaplasia induced by both environmental and genetic factors.

Lastly, we performed single-cell RNA-sequencing analysis on club cells and goblet cells obtained from non-COPD (control) and COPD human lung samples (Watanabe et al., 2022). Pseudotime trajectory analysis demonstrated that club cells act as progenitors of goblet cells, consistent with both our mouse lineage tracing studies and previous findings (Chen et al., 2009). Furthermore, GSEA revealed downregulation of YAP and p53 signatures in goblet cells compared to club cells. These findings indicate a potential role for Hippo and p53 signaling in goblet cell differentiation in human airways. However, whether Hippo cooperates with p53 to directly modulate club to goblet cell differentiation in human airways warrants further investigation.

Overall, our study has identified an essential role for Hippo and p53 in club cell proliferation and differentiation in the airways. These findings provide significant insights into the mechanisms underlying airway epithelial cell fate regulation and mucous cell metaplasia.

## MATERIALS AND METHODS

### Mice

*Scgb1a1-CreERT2*, *Mst1^loxP/loxP^*, *Mst2^−/−^*, *Yap^loxP/loxP^*, *p53^loxP/loxP^* and *Rosa26^tdTomato^* (The Jackson Laboratory, 007914) mouse lines used in this study have been previously described (Dong et al., 2009; Du et al., 2014; Madisen et al., 2010; Marino et al., 2000; Rawlins et al., 2009; Zhang et al., 2010). Mice aged 6-12 weeks of both sexes were used. All mice were kept in specific pathogen-free conditions with a 12 h light/night cycle, an ambient temperature of 20-24°C and 40-60% humidity. All animal experiments were conducted in accordance with protocols approved by the Institutional Animal Care and Use Committee of Shanghai Jiao Tong University.

### LPS-induced airway epithelium injury in mice

Mice were intraperitoneally injected with tamoxifen dissolved in corn oil at a dose of 100 mg/kg body weight/day for five consecutive days to induce gene deletion. Two days after the last dose of tamoxifen injection, the mice received an intratracheal injection of 1 mg/ml LPS (Sigma-Aldrich, L9143) dissolved in 1× PBS at a dose of 1.5 mg/kg body weight. Lungs were harvested 7 days after the LPS treatment.

### PAS staining

We used a PAS staining kit (Solarbio, G1280) for the staining. Paraffin slides were first deparaffinized with xylene, then hydrated in a series of grades of ethanol and finally incubated in water. The tissues on the slides were oxidized with a periodic acid solution for 10 min and then rinsed with water. Next, the tissues were reactivated with Schiff reagent for 15 min and washed with water. They were then stained with a Hematoxylin solution (Solarbio, G1140) for 15 s and washed with water. The tissue slides were transferred to 95% ethanol, further dehydrated with 100% ethanol and xylene, and finally mounted with a neutral balsam medium. The tissue slides were air dried in a chemical hood.

### Alcian Blue and Nuclear Fast Red staining

Alcian Blue solution was prepared by dissolving Alcian Blue 8GX (Sigma-Aldrich, A5268) in a 3% acetic acid solution at a mass/volume ratio of 3%. Paraffin tissue slides were deparaffinized with xylene, hydrated in a series of graded ethanol, and rinsed with water. The tissue slides were then incubated with an Alcian Blue solution for 25 min and washed with water until clear. Subsequently, the tissues were stained with a 2% Nuclear Fast Red solution (Solarbio, G1321) for 3 min and washed with water. The tissue slides were then dehydrated with 95% ethanol, followed by 100% ethanol and xylene. Finally, the slides were mounted with a neutral balsam medium and air dried before imaging.

### Immunostaining

Frozen tissue slides were rehydrated in 1× PBS three times for 10 min each. Paraffin tissue slides were first deparaffinized with xylene, then hydrated with ethanol and finally with water. The tissue slides were then subjected to antigen retrieval by boiling in Tris-EDTA buffer (pH 9.0) using a pressure cooker. Blocking buffer (1× PBS with 3% donkey serum and 0.3% Triton X-100) was added to the tissue slides, and they were blocked for 1 h. After blocking, the tissues were incubated with primary antibodies, diluted in blocking buffer at a ratio of 1:200, overnight at 4°C. The next day, the tissues were washed with 1× PBS three times for 10 min each. The tissue slides were then incubated with secondary antibodies, diluted in 1× PBS at a ratio of 1:500, for 2 h at room temperature. Lastly, the tissues were washed with 1× PBS three times, 20 min each, and mounted with DAPI Fluoromount-G^®^ (SouthernBiotech, 0100-20). Primary and secondary antibodies are listed in Table S1.

### Microscopy imaging

Immunofluorescence images were obtained using a Nikon Ni-E A1 HD25 confocal microscope. PAS and Alcian Blue staining images were acquired by a ZEISS Axio Imager 2 microscope.

### Single-cell RNA-sequencing analysis

The processed single-cell RNA-sequencing data of nine COPD and seven non-COPD (control) samples were downloaded from GSE173896. The data were analyzed using the Seurat package (version 5.0.1) in R (version 4.3.1). The RunPCA function was employed for principal component analysis (PCA), and the top 20 principal components (PCs) were selected with a resolution of 1 for clustering. Clusters of epithelial cells expressing high levels of *EPCAM* and *CDH1* were selected for further analysis. These epithelial cells were then re-clustered using the top 15 PCs and a resolution of 1. Subsequently, cells expressing high levels of club cell marker genes (*SCGB1A1*, *SCGB3A1*, *SCGB3A2*) or goblet cell marker genes (*MUC5AC*, *MUC5B*, *BPIFB1*, *SPDEF*) were selected. These cells were re-clustered with 15 PCs and a resolution of 0.5 to distinguish club cell and goblet cell groups based on their marker gene expression. To perform GSEA, differentially expressed genes (DEGs) between club cells and goblet cells were identified using the FindMarkers function and ranked based on Logfoldchange values. The DEGs were then analyzed by GSEA using the GSEApy package (version 1.0.4) in Python (version 3.9.13). Finally, trajectory analysis was performed using the monocle package (version 2.28.0) in R.

### Statistical analyses

Data are presented as mean±s.d. Statistical significance was determined by unpaired, two-tailed Student's *t*-test calculated with GraphPad Prism 9 (v9.0.0.121). *P*<0.05 was considered statistically significant. All statistical analyses included at least three biological replicates.

## Supplementary Material

10.1242/dmm.052074_sup1 Supplementary information
