# Supplementary material for: Hippo cooperates with p53 to regulate lung airway mucous cell metaplasia
Source: Dis Model Mech. 2024 Nov 18;17(11):dmm052074. doi: 10.1242/dmm.052074 (PMC11603118; doi:10.1242/dmm.052074)
Supplement: Supplementary information [file dmm-17-052074-s1.pdf]

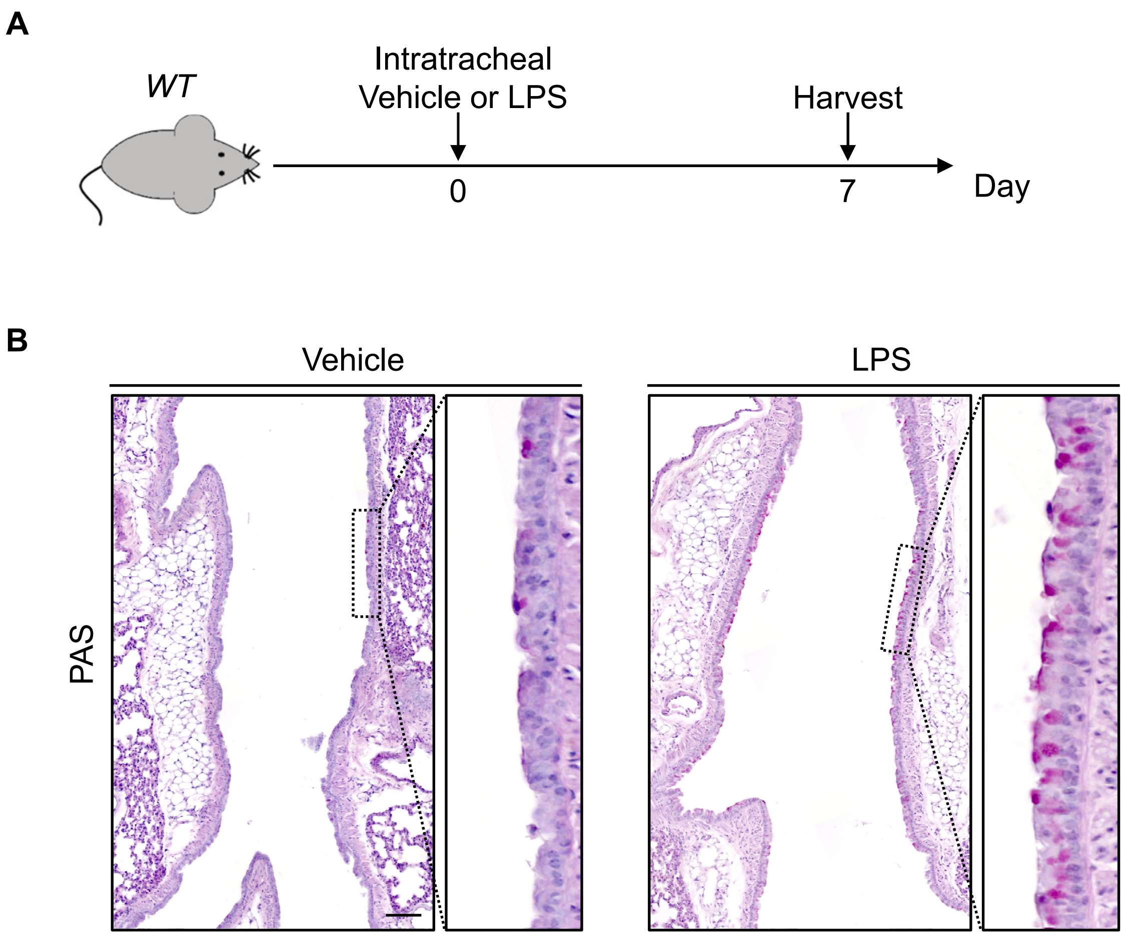


**Fig. S1. LPS induces mucous metaplasia in the airway. (A)** A schematic diagram of the LPS treatment procedure. *WT*, wild type. **(B)** PAS staining of the airway epithelium. Note the increase of PAS$^+$ cells in the LPS-treated airway epithelium. Scale bar: 100 µm.

Disease Models & Mechanisms • Supplementary information

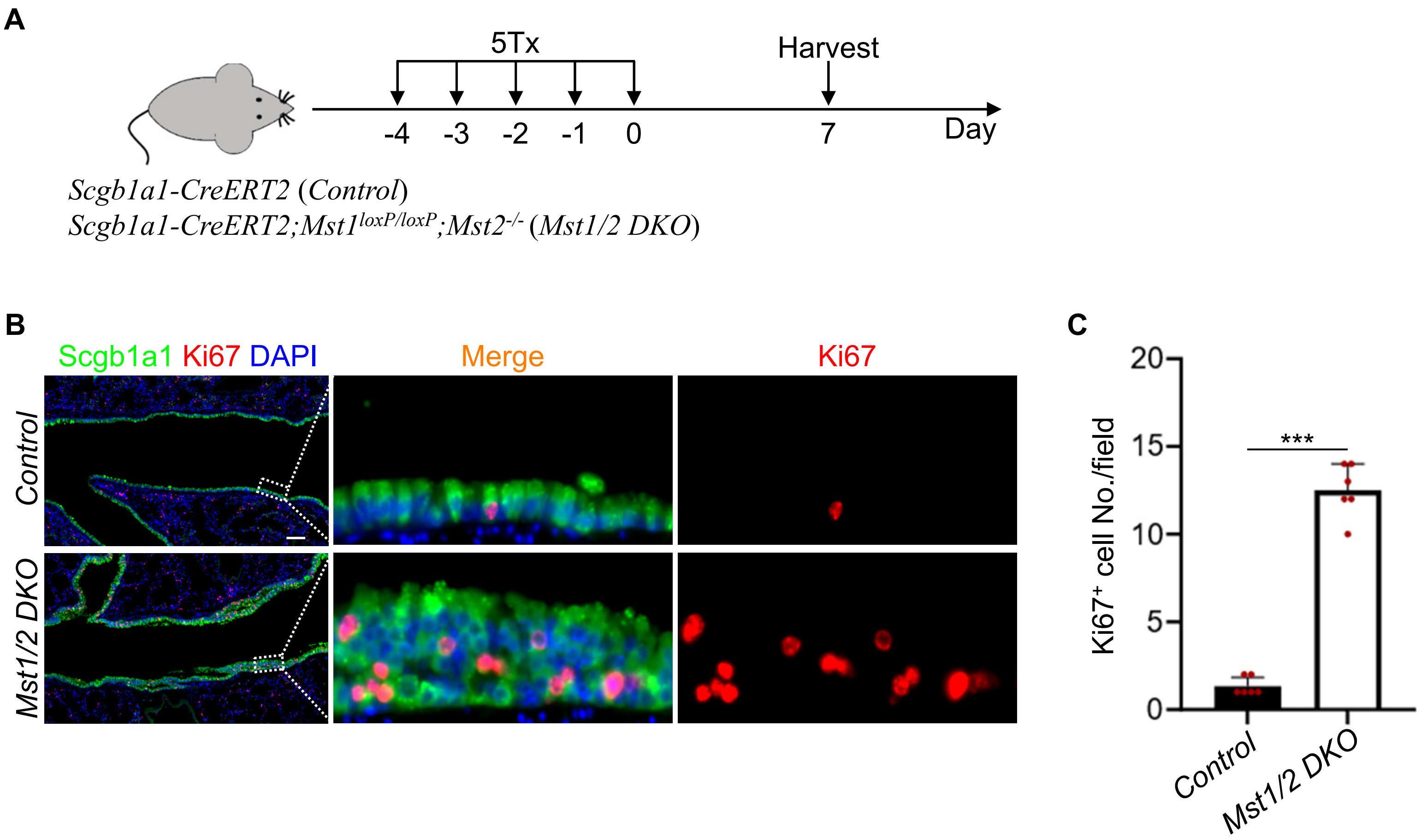


**Fig. S2. Mst1/2 deficiency promotes club cell proliferation in homeostasis. (A)** Schematic diagram of experimental strategies. Tx, tamoxifen. **(B)** Immunofluorescence staining of club cell marker Scgb1a1 and proliferation marker Ki67 in the airway epithelium 1 week after tamoxifen injection. Scale bar: 100 µm. **(C)** Quantification of $Ki67^{+}$ proliferating cells in the airway epithelium. Data represent mean ± SD (n = 6 per genotype). Note the increase in the proliferating cells in *Mst1/2 DKO* mutants. $^{***}p<0.001$, by unpaired, two-tailed Student's *t*-test. *Control, Scgb1a1-CreERT2. Mst1/2 DKO, Scgb1a1-CreERT2;*$Mst1^{loxP/loxP}$*;*$Mst2^{-/-}$.

**Table S1. Antibody list**

| Primary antibody name | Source | Catalog number and RRID | Dilution |
|---|---|---|---|
| Rabbit polyclonal anti-Scgb1a1 | Millipore | Cat#07-623, RRID: AB_310759 | IF: 1:200 |
| Mouse monoclonal anti-Muc5ac | Thermo Fisher Scientific | Cat#MA5-12178, RRID: AB_10978001 | IF: 1:200 |
| Rat monoclonal anti-Ki67 | Thermo Fisher Scientific | Cat#14-5698-82, RRID: AB_10854564 | IF: 1:200 |
| Mouse monoclonal anti-Yap | Santa Cruz Biotechnology | Cat#sc101199, RRID: AB_1131430 | IF : 1:200 |
| Goat Polyclonal anti-tdTomato | Biorbyt | Cat#orb182397, RRID: AB_2687917 | IF : 1:200 |
| Alexa Fluor 488 AffiniPure™ Donkey Anti-Rabbit IgG | Jackson ImmunoResearch Laboratories | Cat#711-545-152, RRID: AB_2313584 | IF: 1:500 |
| Alexa Fluor 488 AffiniPure™ Donkey Anti-Mouse IgG | Jackson ImmunoResearch Laboratories | Cat#715-545-151, RRID: AB_2341099 | IF: 1:500 |
| Alexa Fluor 488 AffiniPure™ Donkey Anti-Rat IgG | Jackson ImmunoResearch Laboratories | Cat#712-545-153, RRID: AB_2340684 | IF: 1:500 |
| Cy™3 AffiniPure™ Donkey Anti-Mouse IgG | Jackson ImmunoResearch Laboratories | Cat#715-165-151, RRID: AB_2315777 | IF: 1:500 |
| Cy™3 AffiniPure™ Donkey Anti-Rat IgG | Jackson ImmunoResearch Laboratories | Cat#712-165-150, RRID: AB_2340666 | IF: 1:500 |
| Cy™3 AffiniPure™ Donkey Anti-Rabbit IgG | Jackson ImmunoResearch Laboratories | Cat#711-165-152, RRID: AB_2307443 | IF: 1:500 |
| Cy™3 AffiniPure™ Donkey Anti-Goat IgG | Jackson ImmunoResearch Laboratories | Cat#705-165-147, RRID: AB_2307351 | IF: 1:500 |
| Cy™5 AffiniPure™ Donkey Anti-Mouse IgG | Jackson ImmunoResearch Laboratories | Cat#715-175-151, RRID: AB_2340820 | IF: 1:500 |
| Cy™5 AffiniPure™ Donkey Anti-Rat IgG | Jackson ImmunoResearch Laboratories | Cat#712-175-153, RRID: AB_2340672 | IF: 1:500 |

Disease Models & Mechanisms • Supplementary information
